# Editorial: Review of hyperbaric therapy & hyperbaric oxygen therapy in the treatment of neurological disorders according to dose of pressure and hyperoxia

**DOI:** 10.3389/fneur.2025.1536541

**Published:** 2025-02-11

**Authors:** Paul G. Harch, George Mychaskiw, John H. Zhang, Dominic P. D'Agostino, Keith Van Meter, Enrico M. Camporesi

**Affiliations:** ^1^School of Medicine, Louisiana State University, New Orleans, LA, United States; ^2^Louisiana State University Health Sciences Center New Orleans, Louisiana State University, New Orleans, LA, United States; ^3^Department of Anesthesiology, Ochsner Louisiana State University Health, Shreveport, LA, United States; ^4^School of Medicine, Loma Linda University, Loma Linda, CA, United States; ^5^Morsani College of Medicine, University of South Florida Health, Tampa, FL, United States

**Keywords:** hyperbaric oxygen therapy (HBOT), hyperbaric therapy, treatment, neurological disorders, pressure, hyperoxia, dosing, brain disorders

## Introduction

Hyperbaric therapy (HT) and hyperbaric oxygen therapy (HBOT) have confused the scientific, medical, and lay communities for 362 years since their first use in England by Nathaniel Henshaw (1662). Therapeutic effectiveness has been attributed to increased barometric pressure for the first 300 years and to increased pressure of oxygen in the modern era. A fortuitous observation by the U.S. Food and Drug Administration in 2011 elucidated the contribution of both barometric pressure and hyperoxia and their bioactivity on a continuum of pressure and hyperoxia. Simultaneous research identified epigenetic activity as a primary mechanism of both pressure and hyperoxia. Given these developments and the drug-like effects of HT and HBOT this Research Topic attracted 13 articles that addressed the dosing of pressure and hyperoxia in HT and HBOT for neurological conditions:

## Acute and chronic pediatric ischemic/hypoxic neurological injury

Mielecki et al. and Sánchez-Rodríguez and López reviewed the animal and human literature on HBOT for perinatal asphyxia and hypoxic-ischemic encephalopathy, concluding that HBOT is safe and more effective than hypothermia in ameliorating or eliminating the sequelae of acute global hypoxic/ischemic insult and subsequent HIE with as little as one rescue treatment administered shortly after birth. Doses in animals were generally at 2.0 ATA and above, while doses in humans tended to be under 2.0 ATA. Marois et al. compared the effectiveness of typical therapies for the common chronic form of pediatric ischemic/hypoxic and hemorrhagic brain injury, cerebral palsy, and demonstrated that HBOT is four times as effective as the average of all other therapies. Using a precise analytical tool the authors found that increased barometric pressure (1.3–1.75 ATA) is the dominant contributor to the effect of HBOT compared to hyperoxia. In light of the science of HBOT in acute HIE and chronic neurological injury, the number of children affected, the devastating consequences, and the impact on quality of life these articles reinforce earlier conclusions that “The time has come”…“for HBOT to be standard of care” [SIC] for these conditions (1).

## Chronic neurological injury

Slade et al. and Jingami et al. reported the first cases of temporally-demonstrated HBOT benefit in chronic stroke thalamic pain syndrome and subacute delayed post-hypoxic leukoencephalopathy (DPHL) secondary to opioid overdose. Post-stroke pain disorders reduce quality of life and affect mood, sleep, and social function. In Slade et al. the authors treated a 55-year-old man who suffered a right thalamic lacunar infarction and developed thalamic pain syndrome. With extensive HBOT over 11 months (100 treatments) the patient's symptoms resolved and his quality of life improved. Jingami et al. reported on the use of acute high-dose HBOT (2.8 ATA oxygen) for suspected carbon monoxide poisoning in a 47-year-old man who was discovered to have overdosed on opioids. Two months later they re-intervened with a dose of HBOT similar to that used by Slade et al. (2.0 ATA oxygen) when DPHL developed. With 62 HBOTs over 140 days, the patient experienced resolution of the neurological symptoms of his ischemic/hypoxic injury. While HBOT can improve cerebral blood flow and metabolism in chronic brain injury this patient's clinical improvement with HBOT raises interesting questions since the improvement occurred despite worsened fronto-parietal ioamphetamine SPECT imaging and evolving cortical atrophy.

## Cancer

Alpuim Costa et al. discussed the use of hyperbaric oxygen therapy (HBOT) as an adjunctive treatment for neuroblastoma, a common pediatric cancer. The authors noted that HBOT reverses tumor hypoxia, a major cause of resistance to traditional treatments such as chemotherapy and radiotherapy, and has other beneficial effects on cancer. Its greatest effect, however, may be modification of the tumor microenvironment (TME) and enhancing the immune response against neuroblastoma cells. Alpuim Costa et al. also reviewed a controlled trial of an HBOT-enhanced IV tumor-specific radioactive compound in children with recurrent Stage IV neuroblastoma, which demonstrated an increase in survival in the HBOT group that was over 2.5 times the control group. The authors recommended future research with HBOT, immunotherapy, and ketone metabolic therapy (KMT) to exploit the unique metabolic vulnerabilities of cancer cells. Wang et al. also discussed the potential of HBOT to enhance cancer immunotherapy through its modification of the TME. HBO enhances radiotherapy and photodynamic therapy by increasing the production of reactive oxygen species and restructuring the extracellular matrix to promote immune cell infiltration. The authors reviewed the effects of HBOT on cancer-specific immune targeting by noting HBOT's ability to improve T cell access to the tumor site, which boosted the effectiveness of immune checkpoint inhibitors such as PD-1/PD-L1 antibodies. Their review of HBOT animal cancer studies showed the effectiveness of HBOT at higher doses in combination with chemotherapy and immunotherapy while HBOT alone had mixed effects on tumor growth.

## Pressure—[diving and altitude (dementia)]

Two manuscripts by Fogarty and Harch and MacLaughlin et al. addressed the second key component of HBOT, barometric pressure, through the application of hyperbaric air. Fogarty and Harch recounted a fascinating history of the bioactivity of low barometric pressure fluctuations, beginning with observations by Evangelista Torricelli in 1644, Henshaw's suggestion of pressurized air as a medical therapy in 1662, O.J. Cunningham's application of pressurized air to Spanish influenza patients in 1918, and M.R. Kramer's successful treatment of COPD patients with 0.14 atmospheres of increased pressurized air in 1996. Fogarty and Harch presented the case of an elderly patient with dementia whose functions were affected by travel involving changes in altitude: deterioration with increasing altitude (decreased pressure) and improvement with decreasing altitude (increased pressure). The authors duplicated the increased pressure benefit with a daily 0.3-atmosphere hyperbaric air treatment (and supplemental glutathione amino acid precursors), which generated a sustained improvement in dementia symptoms. MacLaughlin et al. used the same 0.3-atmosphere increase in air to produce an increase in circulating stem progenitor cells in human subjects. Both papers convincingly suggest that the use of low-dose hyperbaric air as a placebo control in randomized, controlled hyperbaric trials is a treatment in itself, not a control. Simonnet et al., addressed a long-standing debate about the greater contribution of pressure vs. oxygen in the treatment of divers with spinal decompression sickness. The authors' experience suggested that a higher oxygen pressure in the initial treatment is more efficacious than a deeper pressure with less oxygen. This is a provocative finding that does not explain the long-standing U.S. Navy treatment algorithm of converting a failing shallow oxygen table to a deeper pressure table. The findings of Simonnet et al.'s, await replication.

## Psychiatric disease

Andrews and Harch reported a systematic review of HBOT for PTSD. Statistically significant symptomatic improvements were achieved over a wide range of pressures from 1.3 to 2.0 ATA with a linear dose-response relationship between improvement and cumulative oxygen dose. This was accompanied by a severe reversible exacerbation of emotional symptoms at the highest oxygen doses in 30%−39% of subjects, a potential effect of oxygen toxicity. The most surprising findings were imaging abnormalities in PTSD-affected brain regions, suggesting that PTSD should no longer be considered a strictly psychiatric disease.

## Oxygen toxicity

Zaghloul et al. and Harch and Rhodes studied oxygen toxicity across a spectrum of doses from normobaric to hyperbaric oxygen. Zaghloul et al. demonstrated a protective effect of Galantamine administration on mouse pups living in a normobaric hyperoxic environment. Medullary, forebrain, and hippocampal hyperoxia-induced neuronal loss, behavioral/learning/memory deficits, hyaloid artery hyperplasia, retinal cell disruption, and neovascularization were all reduced by daily galantamine administration. These data are highly suggestive of a neuroprotective and ocular protective role for galantamine in oxygen-dependent neonates. In contrast, Harch and Rhodes reported a 20-year experience of acute and chronic central nervous system oxygen toxicity observed during hyperbaric hyperoxic treatment of chronic neurological conditions. Chronic oxygen toxicity was documented at significantly lower pressures over an often extended number of treatments, as measured by a quantitative parameter of cumulative oxygen dose. Multiple clinical observations of hyperoxia were observed before patients attained the parabolic threshold that is documented in the published literature. One case with functional neuroradiological evidence reinforced the significance of these data.

## Conclusion

The above collection of articles has served the purpose of this Research Topic by demonstrating a wide range of bioactivity of hyperbaric pressure and oxygen on disease physiology, pathology, and neurological diseases. The perspective provided will hopefully stimulate additional research into the effects of hyperbaric oxygen and pressure dosage and clinical applications of HBA and HBOT on neurological conditions. It is apparent that after 362 years, the field of hyperbaric medicine is in the infancy of neurological dosing.
